# Genetic Diversity and Population Structure of Urban and Rural Goshawks

**DOI:** 10.1002/ece3.74354

**Published:** 2026-09-11

**Authors:** Jaana A. Kekkonen, Tapio Solonen

**Affiliations:** ^1^ Organismal and Evolutionary Biology Research Programme University of Helsinki Helsinki Finland; ^2^ Luontotutkimus Solonen Oy Helsinki Finland

**Keywords:** *Astur gentilis*, gene flow, genetic structure, microsatellite, urbanization

## Abstract

Urbanization poses a growing threat to biodiversity with potential impacts on species' genetic diversity and population structure. The Eurasian goshawk (*Astur gentilis*) is traditionally a forest‐dwelling raptor that has recently established breeding populations in urban environments such as Helsinki, Finland. Here, we investigated genetic diversity and population structure across urban, suburban, and rural goshawk populations in Finland using 10 microsatellite markers and 72 individuals sampled between 1990 and 2020. Genetic diversity, measured by heterozygosity and allelic richness, was similar among populations. Genetic differentiation was low to moderate (*F*_ST_ = 0.022–0.074) and statistically non‐significant. Despite urbanization, contemporary urban goshawks showed genetic similarity to adjacent contemporary non‐urban goshawks, while greater differentiation was observed between temporally separated populations. Consistent with this pattern, clustering supported *K* = 2 as the primary level of genetic structure, separating the contemporary urban and surrounding populations from the earlier surrounding and rural populations. Given the limited marker set and sample sizes, these findings are interpreted as broad‐scale patterns rather than definitive evidence of fine‐scale population structure. Further studies using larger sample sizes and genome‐wide markers are needed to resolve population connectivity and the longer‐term genetic effects of urbanization.

## Graphical abstract

We investigated genetic diversity and population structure of Eurasian goshawks (*Astur gentilis*) across urban, suburban, and rural habitats in Finland using microsatellite markers. Genetic diversity was similar across groups, while differentiation was generally low, with greater differentiation between temporally separated populations than among contemporary groups. Clustering analyses also provided similar tentative evidence of broad‐scale genetic structure.

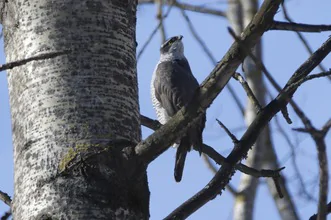

## 1 Introduction

Urbanization is one of the greatest human‐induced threats to biodiversity globally (McKinney 2002; Isaksson 2018). Rapid expansion of human settlements leads to habitat degradation, loss, and fragmentation, thereby increasing barriers to movement of organisms. As the impact of urbanization is expected to increase in the future, it is important to understand the consequences of these processes on species and ecosystems (McKinney 2006; Isaksson 2018). Urbanization provides both challenges and opportunities for species depending on their habitat requirements and sensitivity to changes.

Like many other taxa, bird species have been both failing and succeeding in inhabiting urban areas (Patankar et al. 2021). Birds are very mobile, which can be beneficial as they can utilize larger areas and avoid less favorable ones (Delaney 2014). Birds can also find novel food sources and nesting places in buildings and other infrastructure (Isaksson 2018). However, some bird species move around only in small areas, making them more susceptible to the adverse effects of urbanization (Delaney 2014). Many species also have specific habitat requirements, which may not be met in urban environments (Patankar et al. 2021; Guo et al. 2025).

As urban areas develop, bird populations can become more isolated from populations outside the cities and also more fragmented within the urban areas. This may have long‐term implications for species' survival through impacts on genetics. Maintaining a sufficient level of genetic diversity is important for a species to be able to adapt evolutionarily to changing environments (Frankham 2006; Bruford et al. 2010; Allendorf et al. 2012; Leigh et al. 2019; Shaw et al. 2025). Therefore, monitoring genetic diversity has become one of the priorities in conservation, and management plans should aim to stop the loss of genetic diversity (Hoban et al. 2013, 2020; Laikre et al. 2020). Decreasing genetic diversity can make populations and species vulnerable for example by loss of vital traits, increased risk for inbreeding, decreased reproductive success or increased susceptibility to new pathogens (Leigh et al. 2019, Shaw et al. 2025).

Apart from studying standing genetic variation, it is important to understand population structure. This helps to determine whether there is adequate gene flow between populations to maintain sufficient genetic diversity. Although urban wildlife populations are increasingly studied, the genetic consequences of urbanization remain poorly resolved. Studies have found contrasting patterns ranging from reduced genetic diversity and increased differentiation to little or no genetic change depending on dispersal ability and urban landscape structure (Patankar et al. 2021; Guo et al. 2025). Consequently, it remains unclear whether urban environments primarily act as barriers to gene flow or whether mobile species can maintain genetic connectivity. In urban birds of prey, knowledge on their genetics in urban settings is still relatively limited (Patankar et al. 2021).

The Eurasian goshawk *(Astur gentilis)* is a medium‐sized diurnal bird of prey with a range covering a large part of the Holarctic region from Western Europe through Russia to Japan. In Europe, the species prefers mature forests and has been considered shy and elusive (Kenward 2006). In recent decades, there has been a shift from natural to urban environments, and goshawks have established breeding populations in many cities in Europe like Hamburg or Berlin as well as the Kanagawa area in Japan (Rutz et al. 2006; Rutz 2008; Natsukawa et al. 2019; Solonen et al. 2019; Merling de Chapa et al. 2020). Previous studies have shown behavioral and ecological differences between urban and rural goshawks, including differences in diet and breeding biology (Merling de Chapa et al. 2020; Solonen et al. 2019). Urban populations may benefit from abundant prey resources such as pigeons and corvids but may also experience novel urban‐associated risks (Solonen et al. 2019). Takaki et al. (2009) also found evidence of population genetic structure and moderate genetic differentiation among Eurasian goshawk populations in Japan. However, studies on the genetic diversity of urban goshawk populations remain scarce.

In Finland, the Eurasian goshawk has experienced population declines in recent decades, mainly due to habitat loss and fragmentation associated with forestry (Tornberg et al. 2006; Solonen et al. 2019; Honkala et al. 2024). Goshawk has been protected in Finland since 1982 and is considered as a nearly threatened species (Hyvärinen et al. 2019). Despite being considered an old‐forest species, Finnish goshawks have recently also colonized the Helsinki metropolitan area (Solonen 2026). This population started to increase in the early 2000s and stabilized around 10 years later. Solonen (2026) suggested that increasing food resources like corvids and pigeons may have promoted the colonization of Helsinki.

**FIGURE 1:**
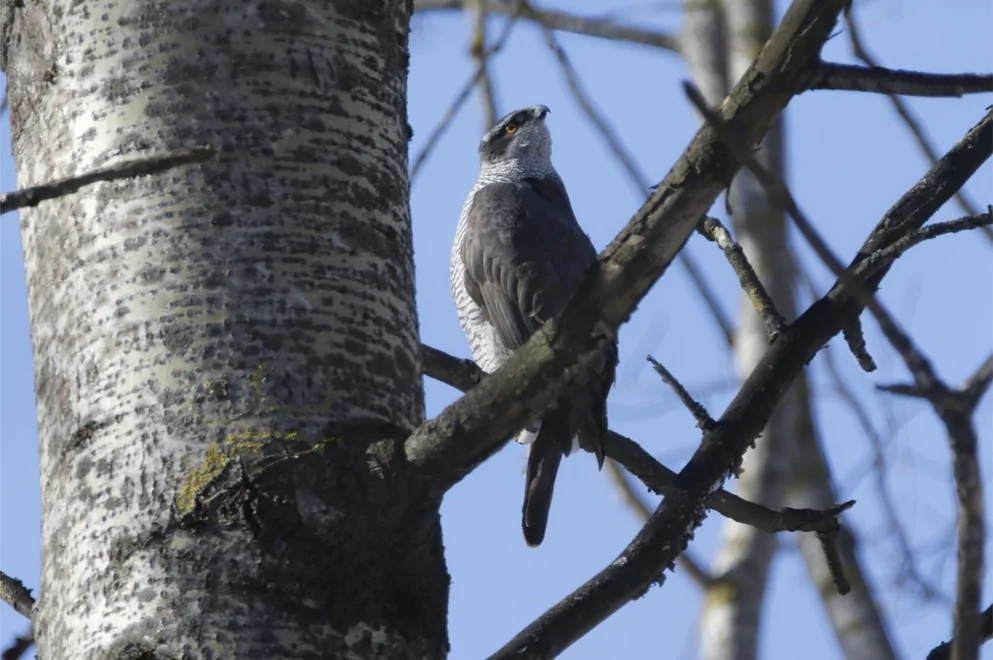
Is the first Finnish urban goshawk population genetically more similar to nearby non‐urban populations than to more distant rural populations? Tapio Solonen.

Here, we investigate the genetic diversity and population structure of goshawks sampled from different environments (urban, near to urban, far from urban) and time periods (past: 1990s and recent: 2019–2020) to assess population differentiation. We hypothesize that urbanization may contribute to genetic differentiation among habitats and potentially reduce genetic diversity in urban populations through habitat fragmentation and altered gene flow. In particular, we examine whether the urban population is genetically more similar to the nearby non‐urban populations than to more distant rural populations (Figure 1).

## 2 Material and Methods

### 2.1 Sampling

**FIGURE 2:**
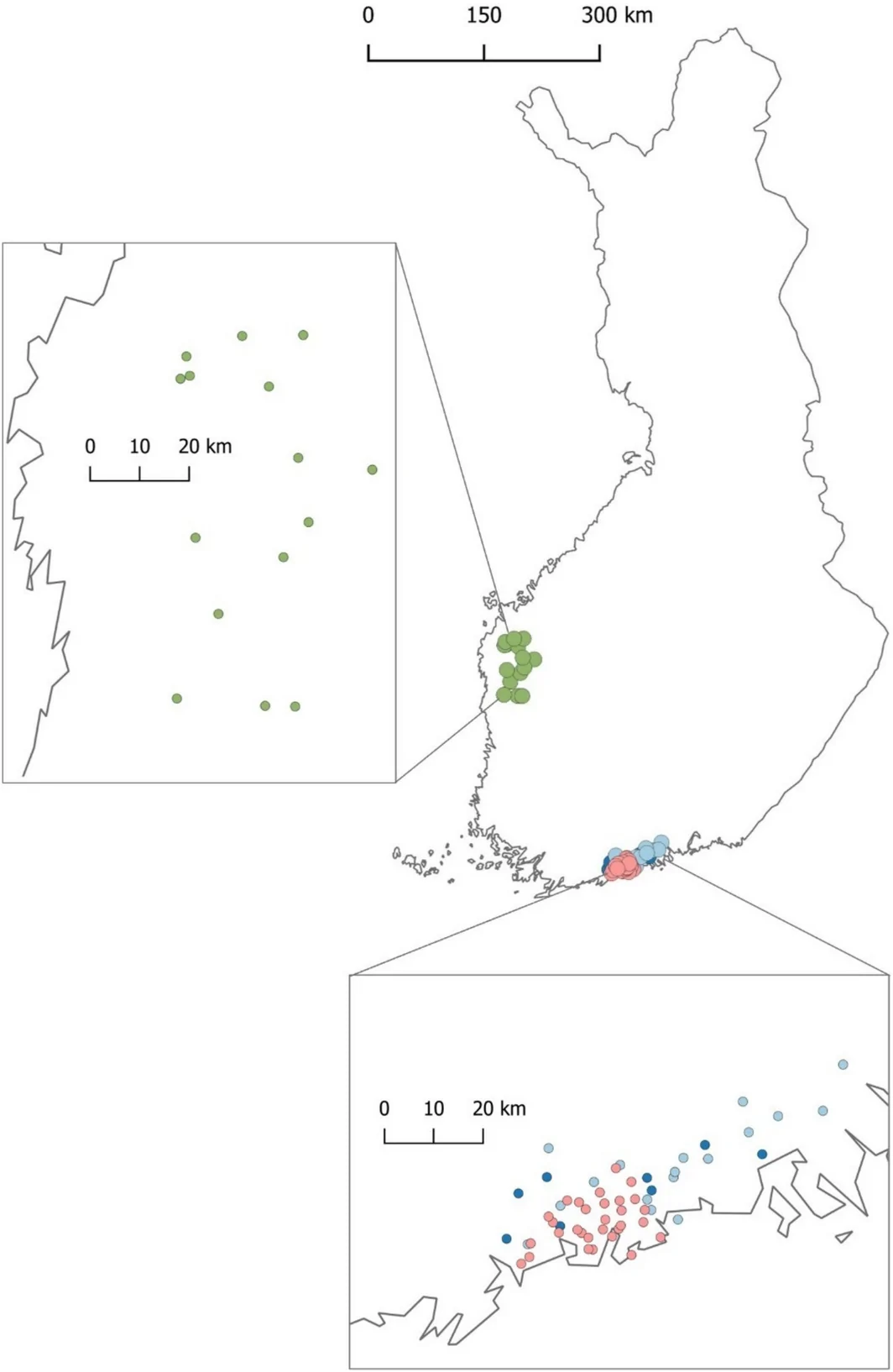
Map of the goshawk sampling locations. Green circles represent rural individuals from Suupohja; pale blue indicate Uusimaa_old; dark blue indicate Uusimaa_new; and pink circles represent urban individuals from the Helsinki district.

Seventy‐two feather samples were collected from nestling goshawks from different broods between 1990 and 2020 in various areas in Southern and Western Finland (Figure 2). We assigned the samples to three groups (urban, suburban, and rural) based primarily on their locality and general environment rather than on a specific habitat type, because the home ranges and foraging strategies of goshawks (Rutz 2006; Väli and Mirski 2026) in our study populations were not specifically known. We then checked the location of each sampling site against the Finnish urban–rural classification developed by the Finnish Environment Institute (SYKE) and the Department of Geography of the University of Oulu (Helminen et al. 2020). The classification is based on geospatial and register data on human population density, land use, spatial distribution of settlements and workplaces, and road networks. The SYKE classification broadly corresponded to our group categorization: our urban samples were located in inner and outer urban areas of the SYKE classification system (classes 1–2, with one peri‐urban site in class 3), suburban samples in peri‐urban and local rural center areas (classes 3–4), and rural samples in rural heartland and sparsely populated rural areas (classes 6–7). Our groups were based on the general environment of the sampling locality, and the SYKE classification was used to characterize the spatial context of each sampling location.

In 1990–1999, samples from 17 individuals were collected from suburban areas around/outskirts of the Helsinki area (Uusimaa_old). In 2019–2020, samples from 30 individuals were collected from urban habitats (Urban) and 9 individuals from suburban habitats (Uusimaa_new) in the Helsinki area. In 1995–1996, samples from 16 individuals were collected from a rural area of Suupohja in Ostrobothnia, Western Finland (Rural). We also used a dataset where we combined Urban and Uusimaa_new into one population and repeated the analyses to assess whether the two should be treated as one genetic population and how this affects the results.

### 2.2 Laboratory Analysis

DNA tissue kit with Chemagic instrument (Revvity) was used to extract DNA from the feathers according to the manufacturer's protocol. Pens of feathers were cut and incubated overnight in lysis buffer and proteinase K before continuing to the extraction in the instrument. Twelve previously developed microsatellites for goshawk were used (Haughey et al. 2016), but two loci were monomorphic in the study population. They were excluded from downstream analyses resulting in a final dataset of 10 polymorphic loci (Table A1). Each PCR reaction contained: 5 μL of Phusion flash master mix (Thermofisher Scientific), 1 μL of both forward and reverse primer, 2 μL of extracted DNA and 1 μL of nuclease‐free water. Following PCR program was used: 98°C for 1 min, 98°C for 1 s, 55°C–62°C for 5 s, 72°C for 15 s, additional extension at 72°C for 2 min. Annealing temperature was 58°C for all primers except 55°C for Age1304 and Age1314 and 62°C for Age1316. Microsatellites were analyzed in an ABI 3730 DNA analyzer (Thermofisher Scientific).

### 2.3 Statistical Analysis

Microchecker 2.2.3 (Van Oosterhout et al. 2004) was used to test for null alleles. The microsatellite loci were also checked for Hardy–Weinberg equilibrium and for linkage disequilibrium with software Genepop 3.4 (Raymond and Rousset 1995). Number of alleles, allelic richness, observed and expected heterozygosities and inbreeding coefficient *F*_IS_ were analyzed with FSTAT 2.9.4 (Goudet 1995). Genetic differentiation among populations was assessed using pairwise *F*_ST_ estimates calculated also in FSTAT v.2.9.4. Significance of pairwise comparisons was assessed using permutation tests based on randomization of individuals among populations (10,000 permutations). *p*‐values were evaluated using a Bonferroni correction for multiple comparisons, resulting in adjusted significance levels of *α* = 0.0083 for comparisons among four populations and *α* = 0.0167 for comparisons among three populations.

Bottleneck program (Piry et al. 1999) was used to see if the populations had experienced a recent reduction in their effective population size. As recommended for microsatellite data, Stepwise mutation model (SMM) and Two‐phase mutation model (TPM) were applied to assess if observed heterozygosity was larger than expected heterozygosity, indicating a recent genetic bottleneck. For the TPM, the proportion of SMM was set to 0.000 and the variance of the geometric distribution to 0.36, corresponding to recommended parameter values. Software Structure 2.2 (Pritchard et al. 2000; Falush et al. 2003, Falush et al. 2007) was used to cluster individuals into groups without prior information on sampling locality (burn‐in period 100,000, Markov chain Monte Carlo repetitions 100,000, simulation was ran 10 times for each value of *K* = 1–5). Software STRUCTURE Selector (Li and Liu 2018) was used to select and visualize the optimal number of clusters from the STRUCTURE results using Puechmaille method (Puechmaille 2016). The Puechmaille method was used instead of the Evanno method because it relies on multiple estimators based on individual assignment probabilities rather than the rate of change in likelihood (ΔK) as in the Evanno method, making it more robust to uneven sample sizes. The method evaluates support for K using four estimators (MedMeaK, MedMedK, MaxMeaK, and MaxMedK) and consistency among these estimators was used to infer the most supported number of clusters. Individuals were also assigned to populations with GeneClass2 software (Piry et al. 2004) using Bayesian criterion by Rannala and Mountain (1997) and the simulation algorithm proposed by Paetkau et al. (2004). MCMC resampling was performed with 10,000 simulated individuals and a *p*‐value threshold of 0.01.

## 3 Results

All 10 microsatellites showed no presence of null alleles when tested with Microchecker. All loci were also in Hardy–Weinberg equilibrium, and no linkage disequilibrium was detected between the loci (Table A1). The 72 samples included in this study had 7 or more microsatellites successfully genotyped (Table A2). The overall proportion of missing genotypes was 11.7% (Rural = 21.9%, Uusimaa_old = 23.5%, Uusimaa_new = 11.1%, Urban = 11.3%).

Genetic diversity was in general similar across the putative populations (Table 1). Mean allelic richness ranged from 2.3 to 2.7, and observed heterozygosity ranged from 0.37 to 0.46. Expected heterozygosity showed a similar pattern. Lowest values were detected in Uusimaa_new. When Urban and Uusimaa_new were combined, heterozygosity values were similar to those of other populations. Over all individuals, mean observed heterozygosity was 0.403 and mean expected heterozygosity was 0.420.

**TABLE 1:** Number of individuals (*N*), mean number of alleles (*N*_a_), allelic richness (*A*_R_), observed heterozygosity (*H*_O_) and expected heterozygosity (*H*_E_), inbreeding coefficient (*F*_IS_) for each sampled population.

Location	Years	*N*	*N*_a_	*A*_R_	*H*_O_	*H*_E_	*F*_IS_ (*p*)
Rural	1995–1996	16	3.3	2.72	0.405	0.476	0.150 (0.053)
Uusimaa_old	1990–1999	17	3	2.58	0.367	0.412	0.109 (0.1676)
Uusimaa_new	2019–2020	9	2.6	2.32	0.366	0.353	−0.037 (0.795)
Urban	2019–2020	30	3.3	2.49	0.459	0.439	−0.047 (0.420)
Urban+UM_new	2019–2020	39	3.5	2.44	0.414	0.421	−0.039 (0.403)

Inbreeding coefficient results from FSTAT software showed a positive *F*_IS_ for Uusimaa_old and Rural (0.109 and 0.150) and a negative *F*_IS_ for Uusimaa_new and Urban (−0.037 and −0.047). For Urban and Uusimaa_new combined, *F*_IS_ = −0.039. Rural population had a nearly significant *F*_IS_ (*p* = 0.053) but none of the other values were statistically significant, providing limited evidence for consistent inbreeding or inbreeding avoidance. Bottleneck software indicated a potential recent bottleneck in all putative populations. A shifted mode was detected for all populations. Wilcoxon two‐tailed tests were significant under both TPM (*p* = 0.002 for all putative populations) and SMM models (*p* = 0.002 for all other putative populations and *p* = 0.004 for Urban). When Urban and Uusimaa_new were combined, the Wilcoxon test was also significant (*p* = 0.002). Moreover, when running the analysis for all individuals as a global sample, a shifted mode was detected and Wilcoxon two‐tailed tests were significant under both TPM (*p* = 0.002) and SMM (*p* = 0.002).

Pairwise *F*_ST_ values ranged between 0.022 and 0.074, indicating low to moderate genetic differentiation among populations (Tables 2 and 3) following the classification of Hartl and Clark (1997). However, the estimates were not statistically significant after correction for multiple testing (*p* = 0.025–0.047, exceeding the adjusted significance threshold α = 0.0083 for four populations and 0.0167 for three populations). For some population pairs, *p*‐values could not be calculated due to limited allelic variation.

**TABLE 2:** Pairwise *F*_ST_ values for four populations.

	Rural	Uusimaa_old	Uusimaa_new	Urban
Rural	—			
Uusimaa_old	0.022	—		
Uusimaa_new	0.050	0.061	—	
Urban	0.056	0.074	0.025	—

**TABLE 3:** Pairwise *F*_ST_ values for three populations, with Urban and Uusimaa_new combined.

	Rural	Uusimaa_old	Urban+Uusimaa_new
Rural	—		
Uusimaa_old	0.022	—	
Urban+Uusimaa_new	0.055	0.067	—

**FIGURE 3:**
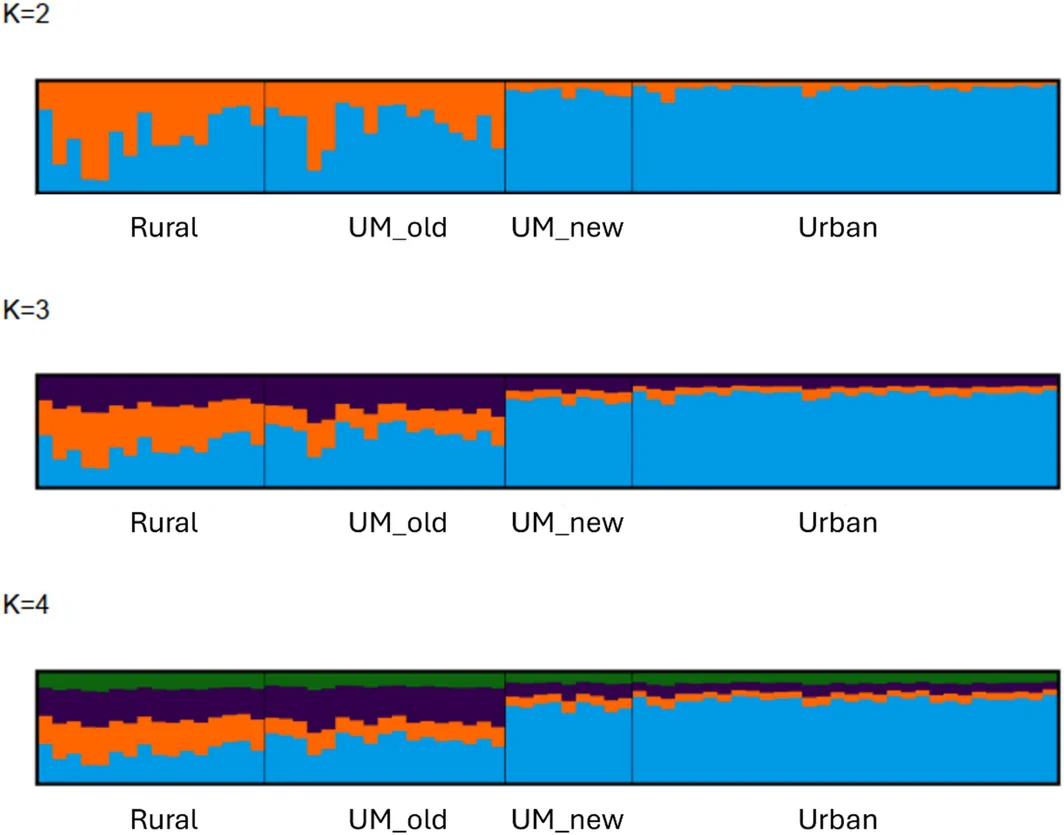
Population Structure Inference Based on STRUCTURE Analysis Visualized by CLUMPAK on four pre‐defined putative populations with *K* = 2, *K* = 3, and *K* = 4.

**FIGURE 4:**
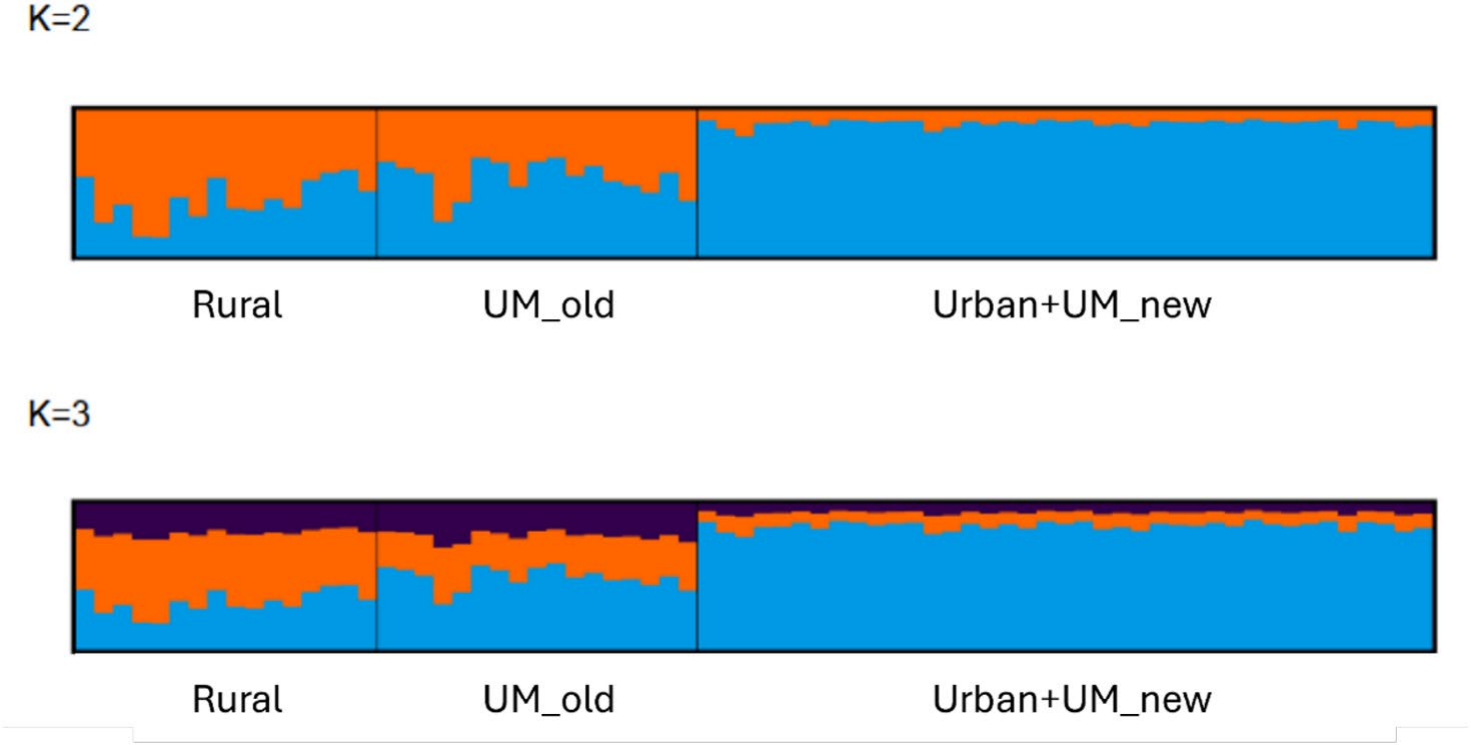
Population Structure Inference Based on STRUCTURE Analysis Visualized by CLUMPAK on three pre‐defined putative populations with *K* = 2 (a) and *K* = 3 (b).

Structure results were consistent with *F*_ST_ values indicating population structuring within the dataset. Post‐analysis using the Puechmaille method (Puechmaille 2016) most strongly supported *K* = 2, both when all putative populations were considered separately and when Uusimaa_new and Urban were combined. *K* = 1 was not supported by any of the estimators. In both population divisions, *K* = 2 represented the primary level of genetic structure, while *K* = 3 was supported by two of the four estimators indicating weaker secondary structure. When four populations were analyzed, MedMeaK and MaxMeaK supported *K* = 2, whereas MedMedK and MaxMedK supported both *K* = 2 and 3 (Figure 3). For the three‐population dataset, MedMeaK and MaxMeaK supported *K* = 2 while MedMedK and MaxMedK supported *K* = 3 (Figure 4). Geneclass 2.0 could assign 38.9% of individuals to populations. When Urban and Uusimaa_new were combined, assignment was 34.7%.

## 4 Discussion

We studied the Eurasian goshawk in Finland to establish the level of genetic diversity and explore how the urban population in the capital area is connected to individuals in suburban and rural settings. Overall, genetic diversity was similar among the sampled populations, and population differentiation was generally low. The largest differentiations were observed between populations sampled in different time periods, suggesting that temporal variation has also contributed to the observed patterns. Clustering analyses suggested some genetic structuring, but these patterns should be interpreted cautiously because the pairwise *F*_ST_ values were not statistically significant.

We found no evidence that urbanization has reduced genetic diversity in the urban goshawk population. This is consistent with previous studies showing that highly mobile raptor species may maintain genetic diversity despite inhabiting urban environments (Rutkowski et al. 2006; Riegert et al. 2010). The similar levels of genetic diversity among habitats may reflect continued gene flow facilitated by the dispersal ability of goshawks (Byholm et al. 2003). Recent work has also highlighted the behavioral flexibility of Eurasian goshawks, demonstrating that individuals can successfully exploit both forest and urban resources (Väli and Mirski 2026). Overall, Finnish goshawks showed genetic diversity broadly comparable to that reported in previous studies, although heterozygosity appeared somewhat lower than in American goshawk (*Astur atricapillus*) populations (Haughey et al. 2016; Sonsthagen et al. 2012) and Eurasian goshawk populations from Japan and other parts of Eurasia (Takaki et al. 2009). However, comparisons among studies should be interpreted cautiously because of differences in marker sets, sample sizes, and study design. Moreover, because the urban population has only recently become established and temporal and spatial variation were partially confounded in the present study, the long‐term genetic consequences of urbanization remain uncertain.

Inbreeding coefficients in the Rural and Uusimaa_old samples were moderate, suggesting some degree of inbreeding (according to Hartl 2000). Although the Rural population showed a marginally non‐significant *F*_IS_ value, results should be interpreted cautiously given the relatively small sample sizes and number of loci. Moreover, the heterogeneous origin of individuals within the Rural sample may have inflated *F*_IS_ estimates. Similar variation in inbreeding coefficients has also been reported among Eurasian goshawk populations in previous studies, where populations in Japan and Russia showed evidence of inbreeding whereas populations in Ukraine and Uzbekistan did not (Takaki et al. 2009). Evidence for recent demographic change was further assessed using bottleneck analyses. Signals of a recent bottleneck were detected in all populations, which could indicate a reduction in effective population size. However, bottleneck analyses based on a limited number of loci and relatively small sample sizes may be prone to false‐positive signals (Peery et al. 2012). Although Finnish goshawk populations have declined considerably during recent decades (Tornberg et al. 2006; Honkala et al. 2024), it remains uncertain whether this demographic decline would be detectable at the genetic level with the marker set and sample sizes used in the present study.

To investigate connectivity between the urban population and surrounding habitats, we examined genetic differentiation among the putative populations. Overall, clustering analyses suggested some population structuring, whereas pairwise *F*_ST_ values indicated only low to moderate and statistically non‐significant differentiation. Together, these results provide only tentative evidence for population subdivision within the Finnish goshawk population. The lowest levels of genetic differentiation were observed between populations sampled during the same time period, whereas the greatest differentiation occurred between populations sampled approximately 20–30 years apart. Similarly, Structure analyses supported *K* = 2 as the primary level of genetic structure, whereas *K* = 1 was not supported. At the primary level, Urban and Uusimaa_new clustered together, while Uusimaa_old was separated despite its close geographic proximity to Uusimaa_new. This pattern suggests that temporal differences in sampling may have contributed to the observed clustering. Because temporal and spatial variation were partially confounded in the sampling design, however, their independent contributions could not be separated. GeneClass assigned 38.9% of individuals correctly to their predefined populations (34.7% when Urban and Uusimaa_new were combined), further supporting the overall weak population structure detected in the dataset. In addition, the limited number of loci, relatively small sample sizes and presence of missing genotypes may have reduced the precision of allele‐frequency estimates and the power of assignment and clustering analyses. Nevertheless, because all individuals were successfully genotyped at a minimum of seven loci, missing data alone are unlikely to account for the weak population structuring observed.

The good dispersal ability of the Eurasian goshawk (median dispersal distance in Finland: 80 km for males and 34.5 km for females; Byholm et al. 2003) is consistent with the low and non‐significant *F*_ST_ between Urban and Uusimaa_new, which were also not separated by the clustering analyses. Thus, there was little evidence that the current level of urbanization has resulted in detectable genetic isolation between urban and adjacent non‐urban areas. The greater differentiation between temporally separated populations, together with the genetic similarity of contemporary samples, suggests a stronger temporal than current spatial component to the observed structure. Nevertheless, the limited number of loci, relatively small sample sizes, and non‐significant *F*_ST_ values mean that these findings should be regarded as preliminary rather than conclusive.

The weak population structuring observed in the present study is broadly consistent with previous goshawk studies, although direct comparisons should be interpreted cautiously. Takaki et al. (2009) likewise reported generally low genetic differentiation among Japanese goshawk populations, despite geographic distances ranging from approximately 100 to 800 km. They also found low to moderate differentiation between Japan and more distant populations from Russia, Ukraine, and Uzbekistan. Similarly, Sonsthagen et al. (2012) reported relatively weak genetic differentiation among North American goshawk populations spanning distances of up to approximately 1200 km. Taken together, these studies suggest that geographic distance alone does not fully explain patterns of genetic differentiation and that local demographic history and landscape characteristics likely also contribute. However, differences in marker sets, population history, habitat characteristics, and sampling design limit direct comparisons.

Overall, our analyses were based on 10 polymorphic microsatellite loci. Although this marker set is suitable for characterizing broad‐scale patterns of genetic diversity and population structure (Koskinen et al. 2004; Arthofer et al. 2018), its resolution is limited for detecting subtle population structure and recent demographic processes. Accordingly, the patterns reported here should be interpreted as broad‐scale trends rather than definitive evidence of fine‐scale population differentiation. Nevertheless, the concordance among different analyses supports the principal patterns identified in this study. Future studies using higher‐resolution genomic data will be needed to resolve these patterns in greater detail.

### 4.1 Future Directions

The decline of goshawks in Finland is alarming, especially given their requirement for relatively large and continuous habitat patches. Urban areas appear to offer novel opportunities, as the species shows some tolerance to human presence and can exploit urban food resources and nesting sites. However, even urban environments may only support goshawks to a limited extent, as observed in the Helsinki district (Solonen 2026). The genetic structure observed in the present study provides an initial indication of connectivity between urban and surrounding populations. Further research using larger sample sizes, replicated urban populations and genome‐wide SNP data would provide greater resolution for assessing population connectivity, demographic history and the effects of urbanization on Finnish goshawks. In particular, sampling contemporary urban, suburban and rural populations would help disentangle the effects of urbanization from temporal variation, which could not be separated in the present study. Combining genomic analyses with ecological and demographic studies would further improve our understanding of how urban environments influence goshawk population viability and breeding performance.

## Author Contributions

**Jaana A. Kekkonen:** conceptualization (equal), data curation (equal), formal analysis (equal), funding acquisition (equal), investigation (equal), project administration (equal), visualization (equal), writing – original draft (equal), writing – review and editing (equal). **Tapio Solonen:** conceptualization (equal), data curation (equal), resources (equal), writing – original draft (equal), writing – review and editing (equal).

## Funding

This work was supported by the Betty Väänänen Foundation.

## Conflicts of Interest

The authors declare no conflicts of interest.

## Supporting information


**Table A1:** Mean number of alleles (*N*_a_), observed heterozygosity (*H*_O_) and expected heterozygosity (*H*_E_) for each microsatellite locus.
**Table A2:** A list of individuals and their genotypes for each locus.

## Data Availability

We provide our data as Tables A1 and A2 at the end of this manuscript.

## References

[ece374354-bib-0001] Allendorf, F. W. , G. Luikart , and S. N. Aitken . 2012. Conservation and the Genetics of Populations. 2nd ed. Wiley‐Blackwell.

[ece374354-bib-0002] Arthofer, W. , C. Heussler , P. Krapf , B. C. Schlick‐Steiner , and F. M. Steiner . 2018. “Identifying the Minimum Number of Microsatellite Loci Needed to Assess Population Genetic Structure: A Case Study in Fly Culturing.” Fly 12, no. 1: 13–22. 10.1080/19336934.2017.1396400. 29166845 PMC5927656

[ece374354-bib-0003] Bruford, M. W. , M. Ancrenaz , L. Chikhi , et al. 2010. “Projecting Genetic Diversity and Population Viability for the Fragmented Orang‐Utan Population in the Kinabatangan Floodplain, Sabah, Malaysia.” Endangered Species Research 12, no. 3: 249–261.

[ece374354-bib-0005] Byholm, P. , P. Saurola , H. Linden , and M. Wikman . 2003. “Causes of Dispersal in Northern Goshawks ( *Accipiter gentilis* ) in Finland.” Auk 120: 706–716.

[ece374354-bib-0006] Delaney, K. S. 2014. “Landscape Genetics of Urban Birds.” In Avian Urban Ecology, 117–128. Oxford University Press.

[ece374354-bib-0007] Falush, D. , M. Stephens , and J. K. Pritchard . 2003. “Inference of Population Structure Using Multilocus Genotype Data: Linked Loci and Correlated Allele Frequencies.” Genetics 164, no. 4: 1567–1587. 12930761 10.1093/genetics/164.4.1567 PMC1462648

[ece374354-bib-0008] Falush, D. , M. Stephens , and J. K. Pritchard . 2007. “Inference of Population Structure Using Multilocus Genotype Data: Dominant Markers and Null Alleles.” Molecular Ecology Notes 7, no. 4: 574–578. 18784791 10.1111/j.1471-8286.2007.01758.x PMC1974779

[ece374354-bib-0010] Frankham, R. 2006. “Genetics and Landscape Connectivity.” In Connectivity Conservation, 72–96. Cambridge University Press.

[ece374354-bib-0012] Goudet, J. 1995. “FSTAT (Version 1.2): A Computer Program to Calculate F‐Statistics.” Journal of Heredity 86, no. 6: 485–486.

[ece374354-bib-0013] Guo, Y. , X. Lu , and Y. Wang . 2025. “Responses of Birds With Different Habitat Preferences to Urban Blue‐Green Spaces: A Systematic Review and Meta‐Analysis at a Global Scale.” Biological Conservation 307: 111,190. 10.1016/j.biocon.2025.111190.

[ece374354-bib-0014] Hartl, D. L. , and A. G. Clark . 1997. Principles of Population Genetics. 3rd ed. Sinauer Associates.

[ece374354-bib-0059] Hartl, D. L. 2000. A Primer of Population Genetics. 3rd ed. Sinauer Associates.

[ece374354-bib-0015] Haughey, C. L. , G. K. Sage , G. R. DeGange , S. A. Sonsthagen , and S. L. Talbot . 2016. “Development of Novel Microsatellite Markers for the Northern Goshawk ( *Accipiter gentilis* ) and Their Utility in Cross‐Species Amplification.” Avian Biology Research 9, no. 3: 195–199.

[ece374354-bib-0016] Helminen, V. , K. Nurmio , and S. Vesanen . 2020. “Kaupunki–Maaseutu‐Alueluokitus 2018: Paikkatietopohjaisen Alueluokituksen Päivitys.” Suomen ympäristökeskus, Helsinki.

[ece374354-bib-0017] Hoban, S. , M. Bruford , J. D. U. Jackson , et al. 2020. “Genetic Diversity Targets and Indicators in the CBD Post‐2020 Global Biodiversity Framework Must Be Improved.” Biological Conservation 248: 108654.

[ece374354-bib-0018] Hoban, S. M. , H. C. Hauffe , S. Pérez‐Espona , et al. 2013. “Bringing Genetic Diversity to the Forefront of Conservation Policy and Management.” Conservation Genetics Resources 5, no. 2: 593–598.

[ece374354-bib-0019] Honkala, J. , P. Lehikoinen , P. Saurola , and J. Valkama . 2024. “Petolintuvuosi 2023—Tunturi‐Lapissa Myyrähuippu, Muualla Ei.” Linnut‐Vuosikirja 2023: 66–77.

[ece374354-bib-0020] Hyvärinen, E. , A. Juslén , E. Kemppainen , A. Uddström , and U. M. Liukko . 2019. Suomen Lajien Uhanalaisuus: Punainen Kirja 2019. Ympäristöministeriö & Suomen ympäristökeskus.

[ece374354-bib-0021] Isaksson, C. 2018. “Impact of Urbanization on Birds.” In Ecology and Conservation of Birds in Urban Environments, edited by D. T. Blumstein , 235–247. Springer.

[ece374354-bib-0022] Kenward, R. 2006. The Goshawk. Poyser.

[ece374354-bib-0023] Koskinen, M. T. , H. Hirvonen , P.‐A. Landry , and C. R. Primmer . 2004. “The Benefits of Increasing the Number of Microsatellites Utilized in Genetic Population Studies: An Empirical Perspective.” Molecular Ecology 13, no. 2: 413–422. 10.1111/j.1601-5223.2004.01804.x. 15383073

[ece374354-bib-0024] Laikre, L. , S. Hoban , M. W. Bruford , et al. 2020. “Post‐2020 goals overlook genetic diversity.” Science 367, no. 6482: 1083–1085. 10.1126/science.abb2748 32139534

[ece374354-bib-0025] Leigh, D. M. , A. P. Hendry , E. Vázquez‐Domínguez , and V. L. Friesen . 2019. “Estimated 6 % Loss of Genetic Variation in Wild Populations Since the Industrial Revolution.” Evolutionary Applications 12, no. 8: 1505–1512. 31462910 10.1111/eva.12810 PMC6708419

[ece374354-bib-0026] Li, Y. L. , and J. X. Liu . 2018. “StructureSelector: A Web‐Based Software to Select and Visualize the Optimal Number of Clusters Using Multiple Methods.” Molecular Ecology Resources 18, no. 1: 176–177. 28921901 10.1111/1755-0998.12719

[ece374354-bib-0028] McKinney, M. L. 2002. “Urbanization, Biodiversity, and Conservation.” Bioscience 52, no. 10: 883–890.

[ece374354-bib-0029] McKinney, M. L. 2006. “Urbanization as a Major Cause of Biotic Homogenization.” Biological Conservation 127, no. 3: 247–260.

[ece374354-bib-0030] Merling de Chapa, M. , A. Courtiol , M. Engler , et al. 2020. “Phantom of the Forest or Successful Citizen? Analysing How Northern Goshawks ( *Accipiter gentilis* ) Cope With the Urban Environment.” Royal Society Open Science 7: 201356. 33489280 10.1098/rsos.201356 PMC7813232

[ece374354-bib-0031] Natsukawa, H. , K. Mori , S. Komuro , T. Shiokawa , J. Umetsu , and T. Ichinose . 2019. “Environmental Factors Affecting the Reproductive Rate of Urban Northern Goshawks.” Journal of Raptor Research 53, no. 4: 377–386.

[ece374354-bib-0034] Paetkau, D. , R. Slade , M. Burden , and A. Estoup . 2004. “Genetic Assignment Methods for the Direct, Real‐Time Estimation of Migration Rate: A Simulation‐Based Exploration of Accuracy and Power.” Molecular Ecology 13, no. 1: 55–65. 14653788 10.1046/j.1365-294x.2004.02008.x

[ece374354-bib-0035] Patankar, S. , R. Jambhekar , K. R. Suryawanshi , and H. Nagendra . 2021. “Which Traits Influence Bird Survival in the City? A Review.” Land 10, no. 2: 92. 10.3390/land10020092.

[ece374354-bib-0036] Peery, M. Z. , R. Kirby , B. N. Reid , et al. 2012. “Reliability of Genetic Bottleneck Tests for Detecting Recent Population Declines.” Molecular Ecology 21, no. 14: 3403–3418. 10.1111/j.1365-294X.2012.05635.x. 22646281

[ece374354-bib-0037] Piry, S. , A. Alapetite , J. M. Cornuet , D. Paetkau , L. Baudouin , and A. Estoup . 2004. “GENECLASS2: A Software for Genetic Assignment and First‐Generation Migrant Detection.” Journal of Heredity 95, no. 6: 536–539. 15475402 10.1093/jhered/esh074

[ece374354-bib-0038] Piry, S. , G. Luikart , and J. M. Cornuet . 1999. “BOTTLENECK: A Computer Program for Detecting Recent Reductions in the Effective Population Size Using Allele Frequency Data.”

[ece374354-bib-0039] Pritchard, J. K. , M. Stephens , and P. Donnelly . 2000. “Inference of Population Structure Using Multilocus Genotype Data.” Genetics 155, no. 2: 945–959. 10835412 10.1093/genetics/155.2.945 PMC1461096

[ece374354-bib-0040] Puechmaille, S. J. 2016. “The Program Structure Does Not Reliably Recover the Correct Population Structure When Sampling Is Uneven: Subsampling and New Estimators Alleviate the Problem.” Molecular Ecology Resources 16, no. 3: 608–627. 26856252 10.1111/1755-0998.12512

[ece374354-bib-0041] Rannala, B. , and J. L. Mountain . 1997. “Detecting Immigration by Using Multilocus Genotypes.” Proceedings of the National Academy of Sciences 94, no. 17: 9197–9201. 10.1073/pnas.94.17.9197 PMC23111 9256459

[ece374354-bib-0042] Raymond, M. , and F. Rousset . 1995. “GENEPOP (Version 1.2): Population Genetics Software for Exact Tests and Ecumenicism.” Journal of Heredity 86, no. 3: 248–249.

[ece374354-bib-0044] Riegert, J. , D. Fainová , and D. Bystřická . 2010. “Genetic Variability, Body Characteristics and Reproductive Parameters of Neighbouring Rural and Urban Common Kestrel (Falco Tinnuculus) Populations.” Population Ecology 52, no. 1: 73–79.

[ece374354-bib-0045] Rutkowski, R. , L. Rejt , and A. Szczuka . 2006. “Analysis of Microsatellite Polymorphism and Genetic Differentiation in Urban and Rural Kestrels *Falco tinnunculus* (L.).” Polish Journal of Ecology 54, no. 3: 473–480.

[ece374354-bib-0046] Rutz, C. 2006. “Home Range Size, Habitat Use, Activity Patterns and Hunting Behaviour of Urban‐Breeding Northern Goshawks *Accipiter gentilis* .” Ardea 94, no. 2: 185–202.

[ece374354-bib-0047] Rutz, C. 2008. “The Establishment of an Urban Bird Population.” Journal of Animal Ecology 77, no. 5: 1008–1019. 18627420 10.1111/j.1365-2656.2008.01420.x

[ece374354-bib-0048] Rutz, C. , R. G. Bijlsma , M. I. C. K. Marquiss , and R. E. Kenward . 2006. “Population Limitation in the Northern Goshawk in Europe: A Review With Case Studies.” Studies in Avian Biology 31, no. 1: 15.

[ece374354-bib-0050] Shaw, R. E. , K. A. Farquharson , M. W. Bruford , et al. 2025. “Global Meta‐Analysis Shows Action Is Needed to Halt Genetic Diversity Loss.” Nature 638, no. 8051: 704–710. 39880948 10.1038/s41586-024-08458-x PMC11839457

[ece374354-bib-0051] Solonen, T. 2026. “Urbanisation of the Eurasian Goshawk (*Astur gentilis*) in Finland.” Ornis Fennica 103: 1–9. 10.51812/of.176803.

[ece374354-bib-0052] Solonen, T. , H. Lokki , and S. Sulkava . 2019. “Diet and Brood Size in Rural and Urban Northern Goshawks ( *Accipiter gentilis* ) in Southern Finland.” Avian Biology Research 12, no. 1: 3–9.

[ece374354-bib-0053] Sonsthagen, S. A. , E. L. McClaren , F. I. Doyle , et al. 2012. “Identification of Metapopulation Dynamics Among Northern Goshawks of the Alexander Archipelago, Alaska, and Coastal British Columbia.” Conservation Genetics 13, no. 4: 1045–1057.

[ece374354-bib-0054] Takaki, Y. , T. Kawahara , H. Kitamura , K. I. Endo , and T. Kudo . 2009. “Genetic Diversity and Genetic Structure of Northern Goshawk ( *Accipiter gentilis* ) Populations in Eastern Japan and Central Asia.” Conservation Genetics 10, no. 2: 269–279.

[ece374354-bib-0055] Tornberg, R. , E. Korpimäki , and P. Byholm . 2006. “Ecology of the Northern Goshawk in Fennoscandia.” Studies in Avian Biology 31, no. 1: 14.

[ece374354-bib-0057] Väli, Ü. , and P. Mirski . 2026. “Diverse Foraging Strategies of an Avian Apex Predator in an Old‐Growth Forest.” Scientific Reports 16, no. 1: 8880. 41787054 10.1038/s41598-026-43036-3 PMC12988056

[ece374354-bib-0058] Van Oosterhout, C. , W. F. Hutchinson , D. P. Wills , and P. Shipley . 2004. “MICRO‐CHECKER: Software for Identifying and Correcting Genotyping Errors in Microsatellite Data.” Molecular Ecology Notes 4, no. 3: 535–538.

