# Supplementary material for: Genetic Diversity and Population Structure of Urban and Rural Goshawks
Source: Ecol Evol. 2026 Sep 11;16(9):e74354. doi: 10.1002/ece3.74354 (PMC13569874; doi:10.1002/ece3.74354)
Supplement: Supplementary file 1 [file ECE3-16-e74354-s001.docx]

**APPENDIX**

Table A1. Mean number of alleles (N_a_), observed heterozygosity (H_O_) and expected heterozygosity (H_E_) for each microsatellite locus

| **Locus** | **N_a_** | **H_O_** | **H_E_** |  |
| --- | --- | --- | --- | --- |
|  |  |  |  |  |
| **Age1302** | 4 | 0.100 | 0.224 |  |
| **Age1303** | 3 | 0.510 | 0.584 |  |
| **Age1304** | 4 | 0.524 | 0.614 |  |
| **Age1305** | 3 | 0.029 | 0.056 |  |
| **Age1308** | 6 | 0.727 | 0.767 |  |
| **Age1312** | 3 | 0.571 | 0.456 |  |
| **Age1314** | 4 | 0.457 | 0.476 |  |
| **Age1316** | 7 | 0.714 | 0.692 |  |
| **Age1317** | 2 | 0.090 | 0.086 |  |
| **Age1321** | 4 | 0.415 | 0.476 |  |

Table A2. A list of individuals and their genotypes for each locus

**Age1302	Age1303	Age1304	Age1305	Age1308	Age1312	Age1314	Age1316	Age1317	Age1321**

pop=Uusimaa_new

308639	150	150	158	160	000	000	148	148	219	223	113	115	120	120	096	096	145	145	120	120

308679	150	150	156	158	000	000	148	148	217	219	115	115	120	120	096	102	145	145	120	120

308682	150	150	158	158	111	111	148	148	215	217	113	113	120	120	094	096	145	145	120	120

314905	150	150	158	158	109	111	148	148	000	000	000	000	120	122	096	096	000	000	118	120

314910	148	150	158	158	107	111	148	148	217	223	113	115	120	120	096	098	145	145	120	120

314913	150	150	156	158	111	111	148	148	000	000	000	000	000	000	096	096	145	145	118	120

314916	150	150	156	158	000	000	148	148	217	217	115	115	120	120	096	098	145	145	120	120

314924	150	150	156	158	107	111	148	148	221	223	113	115	120	122	000	000	145	145	120	120

314927	150	150	158	158	109	109	148	148	217	217	115	117	120	120	096	098	145	145	118	120

pop=Uusimaa_old

900630N	150	150	158	158	107	111	148	148	219	221	113	115	120	122	096	096	145	145	118	120

900630På	148	150	000	000	000	000	000	000	215	219	113	115	120	122	096	096	145	147	118	120

920708S	150	150	000	000	000	000	148	148	000	000	115	115	122	126	096	098	145	145	120	120

970000T	000	000	000	000	107	107	148	148	219	221	115	117	120	120	000	000	145	145	122	122

990624B	148	150	158	158	107	107	148	148	000	000	115	117	120	120	096	096	145	145	120	120

0960630A	150	150	000	000	000	000	000	000	223	223	115	115	122	122	096	098	145	145	120	120

0960630G	150	150	158	158	000	000	148	148	219	223	115	115	000	000	096	096	145	145	120	120

0960630H	150	150	158	158	107	109	148	148	000	000	115	117	000	000	000	000	145	145	120	120

920617O	150	150	158	160	000	000	148	148	217	223	115	115	120	120	098	104	145	145	118	120

0960618I	150	150	158	160	000	000	148	148	217	217	115	115	000	000	000	000	145	145	118	120

0960628V	150	150	158	158	000	000	148	148	221	223	115	115	120	120	000	000	145	145	118	120

0980625E	150	150	000	000	000	000	148	148	219	219	115	115	120	120	000	000	145	145	120	120

920616F	150	150	156	156	000	000	148	148	000	000	115	115	120	126	096	098	145	145	120	120

0980616H	152	152	156	160	107	111	148	148	215	219	000	000	120	122	096	096	145	147	118	118

900621A	000	000	000	000	000	000	148	148	219	221	115	117	120	122	096	104	145	145	000	000

950617P	150	150	000	000	000	000	148	148	215	219	115	115	120	120	096	106	145	145	000	000

920624M	152	152	000	000	000	000	148	148	217	219	115	115	120	126	096	096	000	000	120	120

pop=Rural

S950610Ks	150	150	156	160	107	111	148	148	223	223	113	115	120	120	000	000	145	145	118	118

S950618I	150	150	158	160	000	000	148	150	221	223	113	117	120	126	000	000	145	145	000	000

S950618Lu	150	150	156	160	107	111	148	148	221	223	113	115	120	126	096	104	145	145	120	120

S950618Se	152	152	000	000	107	107	148	152	217	221	000	000	120	126	000	000	145	145	116	118

S950618V	154	154	000	000	107	107	152	152	217	221	115	117	120	120	000	000	145	145	000	000

S950621Pi	150	154	000	000	000	000	148	148	223	223	000	000	120	120	096	096	145	145	120	120

S950624Hu	150	150	000	000	107	107	148	148	217	221	000	000	120	126	000	000	145	145	118	120

S950624Mi	150	150	000	000	000	000	148	148	000	000	113	115	120	120	096	096	145	145	120	120

S950625Kj	150	150	000	000	000	000	148	148	000	000	115	115	120	124	096	106	145	145	120	120

S950625Kn	150	150	156	158	107	113	148	148	000	000	000	000	120	120	104	106	145	145	000	000

S950625So	152	152	156	158	111	111	148	148	000	000	115	115	120	122	000	000	145	145	120	120

S950707Sa	150	150	156	158	107	107	148	148	217	221	000	000	120	120	098	104	145	145	120	120

S950709Jä	150	150	158	158	107	111	148	148	217	223	115	115	120	122	098	106	145	145	120	120

S0960000Ki	150	150	158	158	000	000	148	148	000	000	115	115	120	122	000	000	145	145	118	118

S0960623Km	150	150	156	156	111	111	148	148	219	223	113	115	120	120	098	104	145	145	000	000

S0960624Ha	150	150	000	000	000	000	148	148	000	000	115	115	120	120	094	102	145	145	120	120

pop=Urban

308611	150	150	156	160	111	111	148	148	217	219	115	115	120	120	000	000	145	145	118	118

308626	150	150	156	160	111	111	000	000	215	219	115	115	122	126	094	098	145	145	120	120

308629	150	154	158	158	107	111	148	148	217	225	115	115	122	122	096	104	145	145	118	118

308646	150	150	158	158	107	111	148	148	217	219	113	115	120	120	094	096	145	145	120	120

308650	150	150	160	160	107	109	148	148	217	217	113	115	120	120	000	000	145	147	118	120

314893	150	150	158	160	107	111	148	148	217	217	113	115	120	122	000	000	145	145	118	118

190610E	150	150	156	156	000	000	148	148	219	221	000	000	120	122	096	096	000	000	118	120

308672	150	150	156	158	111	111	148	148	219	223	113	115	120	122	096	096	145	145	118	120

308674	150	150	160	160	111	111	148	148	219	219	115	115	120	120	096	098	145	145	118	120

308684	150	150	000	000	111	111	148	148	219	223	113	115	120	120	098	102	145	147	120	120

308689	150	150	158	158	000	000	148	148	217	223	115	115	120	122	000	000	145	145	118	120

314858	150	150	156	158	000	000	148	148	217	219	113	115	120	120	000	000	145	145	118	118

314861	150	154	156	158	000	000	148	148	215	219	113	115	000	000	096	104	145	145	120	120

314864	150	150	000	000	000	000	148	148	215	223	113	115	120	120	102	106	145	145	118	120

314868	150	150	156	158	107	111	148	148	217	223	113	115	122	122	000	000	145	145	118	120

314872	150	152	158	160	109	111	148	148	217	217	113	115	120	122	098	104	145	145	118	120

314876	150	150	158	160	111	111	148	148	223	223	113	115	120	122	096	104	145	147	118	120

314879	150	150	158	158	107	109	148	148	219	223	113	115	122	122	098	106	145	145	118	120

314883	150	150	160	160	109	111	148	148	217	217	113	115	120	122	096	098	145	145	120	120

314887	150	150	000	000	109	111	148	148	217	221	113	115	122	122	000	000	145	145	118	118

314888	150	150	000	000	111	111	148	148	000	000	115	115	120	122	096	096	145	145	118	120

314889	150	150	000	000	107	113	148	148	219	219	115	115	120	122	096	098	145	145	118	120

314897	150	150	000	000	111	113	148	148	000	000	115	115	120	122	000	000	145	145	118	120

314899	150	150	158	158	000	000	148	148	215	217	113	115	120	120	100	104	145	145	120	120

314900	150	150	158	158	000	000	148	148	217	217	113	115	120	120	096	104	145	145	118	120

314901	150	150	000	000	000	000	148	148	215	217	113	115	120	122	098	102	145	145	000	000

314907	150	150	156	158	000	000	148	148	000	000	113	115	120	120	000	000	145	145	120	120

314919	150	150	156	158	109	111	148	148	219	223	113	115	120	122	096	098	145	147	118	120

314920	150	150	000	000	111	111	148	148	219	223	113	115	120	120	096	100	145	145	120	120

314921	150	150	156	158	111	111	148	148	217	217	113	115	120	122	096	096	145	145	118	120
